# Antioxidant activities of two sericin proteins extracted from cocoon of silkworm (*Bombyx mori*) measured by DPPH, chemiluminescence, ORAC and ESR methods

**DOI:** 10.3892/br.2014.244

**Published:** 2014-02-28

**Authors:** TAYORI TAKECHI, RITSUKO WADA, TSUBASA FUKUDA, KAZUKI HARADA, HITOSHI TAKAMURA

**Affiliations:** 1 Faculty of Human Science, Kobe Shoin Women’s University, Hyogo 657-0015, Japan; 2 Graduate School of Humanities and Science, Nara Women’s University, Nara 630-8506, Japan; 3 Laboratory of Marine Food Processing and Safety, Department of Food Science and Technology, National Fisheries University, Independent Administrative Institution, The Ministry of Agriculture Forestry and Fisheries of Japan, Yamaguchi 759-6595, Japan; 4 KYOUSEI Science Center for Life and Nature, Nara Women’s University, Nara 630-8506, Japan

**Keywords:** 1,1-Diphenyl-2-picrylhydrazyl, electron spin resonance, antioxidant activity, sericin, resistant protein, chemiluminescence, oxygen radical absorbance capacity

## Abstract

Recent efforts have focused on the use of sericin proteins extracted from cocoons of silkworm as a healthy food source for human consumption. In this study, we focused on the antioxidative properties of sericin proteins. The antioxidative properties were measured in sericin proteins extracted from the shell of the cocoon, designated hereafter as white sericin protein and yellow-green sericin protein, as well as bread without sericin protein and bread to which white sericin powder had been added using four measurement methods: 1,1-Diphenyl-2-picrylhydrazyl (DPPH), chemiluminescence, oxygen radical absorbance capacity (ORAC) and electron spin resonance (ESR). High antioxidative properties of sericin proteins were indicated by all four methods. A comparison of the two types of sericin proteins revealed that yellow-green sericin protein exhibited high antioxidative properties as indicated by the DPPH, chemiluminescence and ORAC methods. By contrast, a higher antioxidative property was determined in white sericin protein by the ESR method. Consequently, our findings confirmed that sericin proteins have antioxidative properties against multiple radicals. In addition, the antioxidative property of bread was enhanced by the addition of sericin powder to the bread. Therefore, findings of this study suggest that sericin proteins may be efficiently used as beneficial food for human health.

## Introduction

Sericin protein from the cocoon of silkworm (*Bombyx mori*) is regarded as the resistant protein against digestion together with buckwheat and soybean proteins (1–3). Sericin is a type of protein created by silkworms in the production of silk. Silk emitted by the silkworm consists of two main proteins, sericin and fibroin, with fibroin being the structural center of the silk, and sericin being the sticky material surrounding it. Notably, during the production of silk cloth, the sericin protein is discarded.

In a previous study, we reported the dyeing property of silkworm cocoon (4,5). Lipid oxidation on vegetable oil and frying oil using the indicators peroxide value (POV), carbonyl value (COV) and anisidin value (AnV) has been previously identified (6,7). Additionally, recent studies have focused on the antioxidative activity of seafood using oxygen radical absorbance capacity (ORAC), hydroxyl radical averting capacity (HORAC) and electron spin resonance (ESR) methods (8,9).

By contrast, Dash *et al* showed that sericin protein from silkworm inhibited UVB-induced apoptosis in human skin keratinocytes (10), and that the antioxidant potential of the protein against hydrogen peroxide-induced oxidative stress in skin fibroblasts using the indicators of catalase, lactate dehydrogenase and malondialdehyde activities (11). Zhaorigetu *et al* demonstrated that sericin protein exerts a suppressant effect on skin cancer (12) and colon cancer (13), while Li *et al* demonstrated a protective effect of sericin and sericin peptide against alcohol-induced liver and gastric injuries in mice (14,15). Aramwit *et al* (16) and Ohnishi *et al* (17) identified a cell-protective effect of sericin on collagen and nitric oxide, and cryopreserved rat islets, respectively. Additionally, Manosroi *et al* (18) and Jin-Bo *et al* (19) identified the antioxidant and tyrosinase inhibition activity of sericin protein extracted from silkworm.

Efforts have been made to determine whether the utilization of sericin proteins extracted from cocoon of silkworm (*Bombyx mori*) may be beneficial for human consumption. One of the physico-chemical properties of sericin is its antioxidant activity (20). Cocoon shells vary in color, i.e., white, green, yellow and red. The color components coexist and accumulate in the sericin layer of cocoons. Among different colored cocoons, yellow-green cocoon shells contain various flavonol compounds with antioxidant activity (21). In this study, we investigated the antioxidant activities of different types of sericin proteins extracted from the shell of the cocoon, designated hereafter as white sericin protein and yellow-green sericin protein, using the 1,1-Diphenyl-2-picrylhydrazyl (DPPH), chemiluminescence, ORAC and ESR methods. Furthermore, we examined whether or not the antioxidant activity of bread was elevated by the addition of sericin protein.

## Materials and methods

### Samples

White sericin and yellow-green sericin powder was used. Sericin powder was also added to bread, while additive-free bread served as the control. White sericin powder was purchased from Kashiro Sangyo Co. Ltd. (Shiga, Japan) and yellow-green sericin powder was purchased from Shiono-ya (Kyoto, Japan). Bread was used as an example of processed food to which sericin powder was added.

### Preparation of the bread

Ingredients including wheat flour (Camellia, Nissin Flour Milling, Tokyo, Japan), sericin, dry yeast (Super Camellia, Nissin Foods Group, Tokyo, Japan) and butter (Yotsuba Milk Products Co., Ltd., Hokkaido, Japan) were placed in a bread machine (National Panasonic, SD-BM101, Osaka, Japan) (Table I). Bread was made in the preset standard course (4 h).

### Sample preparation

To measure the antioxidant activity, each sample (5 g) was homogenized with 5 ml water using a mortar for 2 min. Then, 15 ml of water was added and the mixture was homogenized for 1 min. The sample solutions were centrifuged at 15,000 × g for 15 min. The supernatant was removed and the sample was frozen at −30°C as the stock sample solutions.

### Chemicals

DPPH, tris (hydroxymethyl) aminomethane (Tris), 2,2′-Azobis (2-amidinopropane) dihydrochloride (AAPH), potassium dihydrogenphosphate (KH_2_PO_4_), dipotassium hydrogenphosphate (K_2_HPO_4_), ethanol (99.5%), methanol (HPLC grade), hydrochloric acid, sodium dihydrogenphosphate (NaH_2_PO_4_), disodium hydrogenphosphate (Na_2_HPO_4_) and sodium tetraborate were purchased from Wako Pure Chemical Industries, Ltd. (Osaka, Japan). Fluorescein sodium salt was purchased from Sigma-Aldrich Japan (Tokyo, Japan), and 6-hydroxy-2,5,7,8-tetramethyl-chroman-2-carboxylic acid (Trolox) was purchased from Tokyo Kasei Kogyo Co., Ltd. (Tokyo, Japan). Luminol and cytochrome *c* from horse heart were obtained from Nacalai Tesque (Kyoto, Japan).

### Measurement of DPPH radical scavenging activity

Measurement of DPPH radical scavenging activity for all fractions was performed using the method by Yamaguchi *et al* (22). Briefly, an aliquot of antioxidant solution (200 μl) was mixed with 100 mM Tris-HCl buffer (pH 7.4, 800 μl) and added to 1 ml of 500 μM DPPH in EtOH. The mixture was agitated vigorously, left to stand for 20 min at room temperature in the dark, and then subjected to reverse-phase high performance liquid chromatography (HPLC) analysis. The HPLC equipment consisted of a Shimadzu LC-6A pump, a Rheodyne injector fitted with a 20 μl loop and a Shimadzu SPD-6AV UV-VIS detector set at 517 nm (0.064 AUFS). Analyses were conducted in a TSKgel Octyl-80Ts column (4.6×250 mm, Tosoh, Tokyo, Japan) at room temperature with a mobile phase of MeOH/H_2_O (7:3, v/v) at a flow rate of 0.8 ml/min. The DPPH radical-scavenging activity was evaluated from the difference in peak area decrease of the DPPH radical detected at 517 nm between a blank and a sample. Trolox was used as a control standard. The data were expressed as mmol of Trolox equivalent per 100 ml of each sericin sample.

### Chemiluminescence experiment procedure

The chemiluminescence method has been described in detail previously (23). Briefly, the AAPH (40 mM) reagent was dissolved in 100 mM phosphate buffer (pH 7.0). The sericin sample solutions were also diluted to the appropriate concentration using the same buffer. The AAPH solution was heated at 37°C for 2 min to generate peroxyl radicals. The AAPH solution (0.2 ml) was then mixed with 0.2 ml phosphate buffer as the control or with 0.2 ml diluted sericin sample, and the solution mixture was then heated at 37°C for 2 min. For exposure to heating, a water bath (Thermo Max TM-1, AS ONE Corporation, Osaka, Japan) was used. The temperature was maintained within ±3°C. Immediately after heating, 0.2 ml luminol solution was added to the mixture for chemiluminescence measurement. For the luminol solution, luminol (0.113 mM) and cytochrome *c* (0.004 mM) were dissolved in a mixture of 100 mM sodium tetraborate buffer (pH 9.28), water and methanol (volume ratio, 9:1:30). Final concentrations of AAPH, luminol and cytochrome *c* were 13.333, 0.038 and 0.001 mM, respectively. Chemiluminescence intensity was measured using a photon counter Lumitester C-110 (Kikkoman Co., Tokyo, Japan), where one relative light unit (RLU) represented ~43 photons/sec.

### Calculation of the IC_50_ of peroxyl radical scavenging

As an indicator of antioxidative capacity, the inhibition of chemiluminescence intensity was measured by the change of RLU value. The lower the RLU value the more inhibition of chemiluminescence intensity. The value of IC_50_ was defined as the concentration of the sericin sample that reduced the RLU value of the phosphate buffer (control) to half. First, the antioxidative value was calculated using the formula: (log Io/I) × 100, where Io is the RLU value of the phosphate buffer as the control, and I is the RLU value of each concentration of the sericin sample. When the value of this formula was 30.103, the I value corresponded to half-inhibition.

### Preparation of hydrophilic-oxygen radical absorbance capacity (H-ORAC) reaction solution

H-ORAC reaction solution was prepared as previously described (9). Briefly, phosphate buffer was used as the assay (control) buffer and was prepared by combining 75 mM K_2_HPO_4_ and 75 mM KH_2_PO_4_ to a final volume of 75 μmol (pH 7.0). The AAPH reagent was dissolved in the buffer at a concentration of 31.7 mM. Fluorescein working solution was prepared at a concentration of 94.4 nM by dissolving fluorescein sodium salt in the buffer. Trolox standard solutions were prepared at concentrations of 100, 50, 25, 12.5 and 6.25 μM by dissolving Trolox in the buffer.

### Measurement of H-ORAC

The ORAC value was obtained by measuring the elimination capacity of peroxyl radicals generated by the AAPH reagent and by measuring the time lapse degradation of fluorescein (i.e., the rate of decrease in the intensity of fluorescence). The ORAC assay was performed using a 96-well Mithras LB940 multimode microplate reader (Berthold Technologies GmbH & Co. KG, Bad Wildbad, Germany) as previously described (9). Briefly, 20 μl of each sample buffer (obtained by appropriate dilution with the assay buffer) and various concentrations of trolox standard solution (for concentration of a standard curve) or blank buffer (as a control) were placed in the individual wells of a 96-well transparent microplate (Sanplatec Corp., Osaka, Japan). Fluorescein working solution (200 μl) was added and the wells were agitated at 37°C for 10 min. Subsequently, 75 μl of AAPH solution was added to each of the wells to initiate the reaction. The total volume of each reaction solution was 295 μl. The fluorescence intensity [485 nm (excitation)/535 nm (emission)] was then measured every 2 min over 90 min at pH 7.4 and 37°C. As the reaction progressed, fluorescein was consumed and the fluorescence intensity was decreased. The inhibition of fluorescence decay was considered to indicate the presence of an antioxidant.

Typical ORAC assay kinetic curves in the presence of various concentrations of trolox were determined. ORAC values were then measured. The area under the kinetic curve (AUC) of the standards and samples was calculated as: AUC = (0.5 + f_10 min_/f_8 min_ + f_12 min_/f_8 min_ + f_14 min_/f_8 min_ + ··· + f_90 min_/f_8 min_) × 2, where f_x min_ is the fluorescence reading at cycle × min (23).

The standard regression line was obtained by plotting the trolox concentrations against the net AUC_trolox_ of each concentration: Net AUC_trolox_ = AUC_trolox_ − AUC_control_, and Net AUC_sample_ = AUC_sample_ − AUC_control_; where AUC_trolox_ is the AUC in the presence of trolox; AUC_control_ is the AUC with blank control and AUC_sample_ is the AUC with sample buffer. The horizontal axis is the net AUC_trolox_ and the vertical axis is the concentration of trolox. The equation Y = ax + b was derived from the above data and the values for a and b were obtained.

The final ORAC values of the samples were calculated using the equation: ORAC value (μmol trolox equivalent/100 g) = [a × (net AUC_sample_)] × 100/[sample], where [sample] is the diluted concentration ratio of the sample. Data were presented using Microsoft Excel.

### Electron spin resonance analysis

ESR was conducted as previously described (8). Briefly, hydroxyl radical generation was first examined by the DMPO method and iron (II) sulfate with or without the fish sauce samples. The addition of 8.8 mM H_2_O_2_ (50 μl) to the reaction mixture (320 μl) was used to initiate Fenton’s reaction as depicted in the chemical equation: Fe^2+^ + H_2_O_2_ → Fe^3+^ + ·OH + OH^−^. After l min of hydroxyl radical generation, spin adduct DMPO-OH· was measured using the ESR spectrometer (JES-FR30: JEOL Ltd., Tokyo, Japan). ESR measurement conditions were: output, 4 mW (9.4 GHz); magnetic field, 342.790±5 mT; modulation amplitude, 0.079 mT; time constant, 0.1 sec; sweeping time, l min; amplification ratio, 32–125.

### Calculation of the IC_50_ of hydroxyl radical scavenging

IC_50_ values were defined as the concentration of each sericin sample that reduced the control peak height ratio of ESR (generation of hydroxyl radical) by half. The antioxidative value was calculated using the formula: (log Io/I) × 100, where Io is the ESR peak height ratio as the control, and I is the ESR peak height ratio as a sample. Thus, the IC_50_ value was the concentration of samples at Io/I = 1/2, calculated from the antioxidant results of ESR obtained in the experiments.

### Statistical analysis

The Fisher’s exact test, Student’s t-test and correlation analysis were performed using Microsoft excel.

## Results and Discussion

### Measurement of radical scavenging activity by the DPPH-HPLC method

Measured results are shown in Table II. DPPH is a stable, artificial-free radical. Yamaguchi *et al* reported that the DPPH radical scavenging activities of onion, broccoli, and burdock were 104, 890, and 3685 μmol Trolox eq./100 g, respectively (24,25). When compared with these values, yellow-green and white sericin proteins were 23.7 and 18.2 mmol Trolox eq./100 g, respectively, indicating high DPPH radical scavenging activities. The value of yellow-green sericin was significantly higher than that of white sericin. Higher DPPH radical scavenging activity was presumably caused by the additional effect of flavonoid pigment contained in yellow-green sericin. Thus, sericin proteins may demonstrate a high DPPH radical scavenging activity.

### Measurement of peroxyl radical scavenging activity by chemiluminescence method

**Figure 1:**
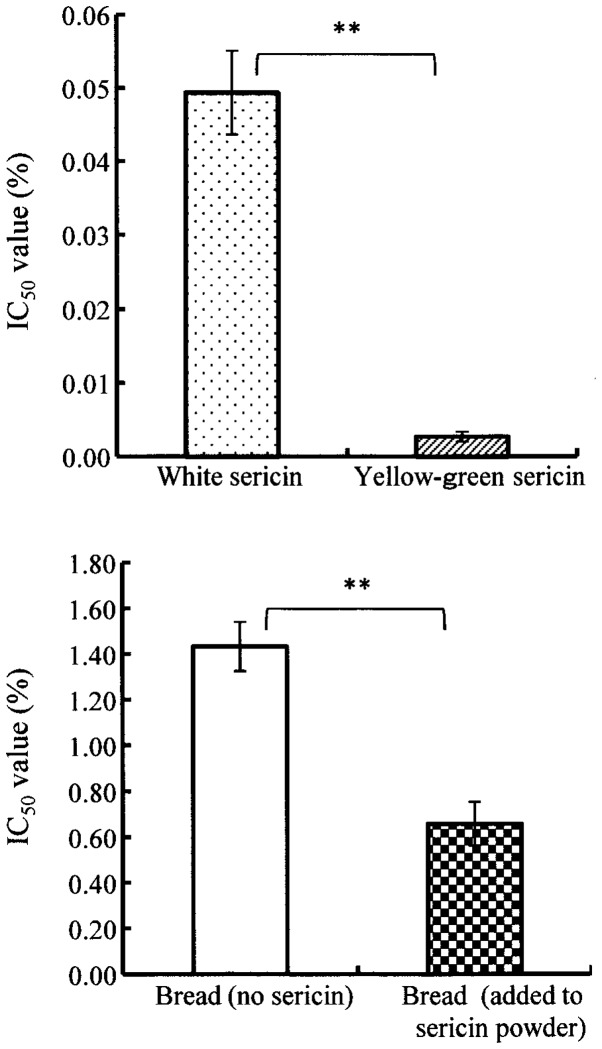
Antioxidant activity of sericin proteins measured by chemiluminescence. Mean ± SD (n=3); ^**^P<0.01.

Measured results are shown in Fig. 1. The yellow-green and white sericin proteins indicated high peroxyl radical scavenging activities. Nagao *et al* reported that the IC_50_ value of the peroxyl radical scavenging activity was 0.1% in kale, which is known to exhibit a high antioxidative property (water-extracted sample) (26). Specifically, the antioxidant activity of yellow-green sericin protein < 0.01% of the IC_50_ value was significantly higher than that of the white sericin protein of ~0.05% of IC_50_ value (p<0.01). In the same manner as the DPPH radical scavenging activity, it is considered that the higher peroxyl radical scavenging activity was caused by additional effect of flavonoid pigment contained in yellow-green sericin. In addition, bread with sericin powder had significantly high peroxyl radical scavenging activity when compared with bread without sericin powder (p<0.01). Improvement of peroxyl radical scavenging activity was observed by the addition of sericin (~4% of bread powder).

### Measurement of peroxyl radical eliminating activity by the ORAC method

As shown in Table III, the peroxyl radical eliminating activity was measured by the ORAC method. It has been reported by Yamaguchi *et al* (25) that peroxyl radical eliminating activities of burdock, ginger, and komatsuna (Japanese mustard spinach) were 7.5, 4.8, and 12.3 mmol Trolox eq./100 g, respectively, and the activity of kamaboko was also reported by Harada *et al* (9) as 0.166 mmol Trolox eq./100 g. Particularly, the value of yellow-green sericin protein (29.3 mmol Trolox eq./100 g) was significantly higher than that of the white sericin protein (10.0 mmol Trolox eq./100 g) (p<0.05). Similar to the DPPH and peroxyl radical scavenging activities, higher peroxyl radical eliminating activity was presumably the result of flavonoid pigment contained in yellow-green sericin protein.

### Measurement of hydroxyl radical scavenging activity by the ESR method

**Figure 2:**
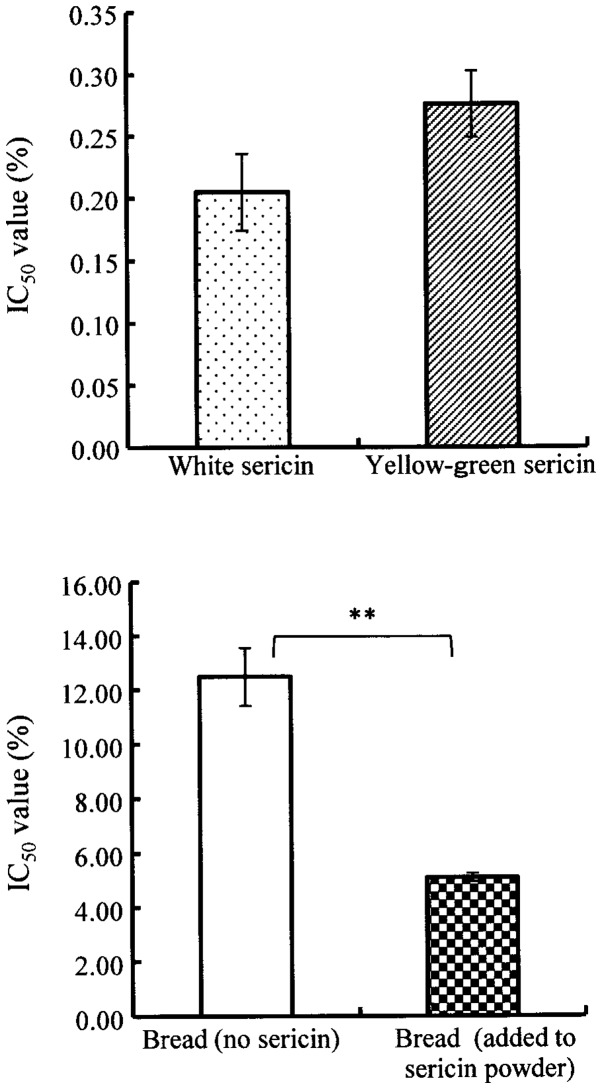
Antioxidant activity of sericin proteins measured by the ESR method. Mean ± SD (n=3); ^**^P<0.01. ESR, electron spin resonance.

As shown in Fig. 2, the hydroxyl radical scavenging activity was measured by the ESR method. The yellow-green and white sericin proteins indicated high antioxidant activity wherein the activity of white sericin protein was higher than that of yellow-green sericin protein although there was no significant difference. By using all the above-mentioned methods, yellow-green sericin indicated higher values. Based on these results, it is assumed that the antioxidant capacity of flavonoid pigment may not be involved in the elimination of hydroxyl radicals. Additionally, bread with sericin powder had significantly high hydroxyl radical scavenging activity when compared with bread without sericin. As for the hydroxyl radical scavenging activity, the improvement of hydroxyl radical scavenging activity was observed by the addition of sericin (~4 % of bread powder) (Fig. 2).

### Correlation between DPPH radical scavenging activity and ORAC value

**Figure 3:**
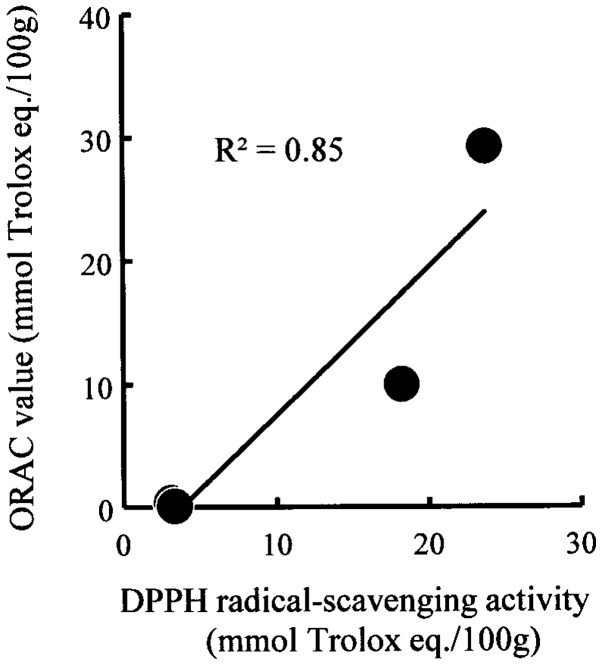
Comparison of the DPPH radical scavenging activity with the ORAC value of sericin proteins. DPPH, 1,1-Diphenyl-2-picrylhydrazyl; ORAC, oxygen radical absorbance capacity.

Based on the values obtained from the four types of samples, the correlation coefficient between the DPPH radical scavenging activity and ORAC value was calculated (Fig. 3). As shown in Fig. 3, a positive correlation of R^2^=0.85 was observed. Conflicting reports have concluded that DPPH and ORAC methods have no correlation (27) or that the methods are correlated (28). By contrast, a positive correlation was observed in the case of sericin proteins and bread to which sericin powder was added.

### Antioxidant property of sericin proteins

Antioxidative mechanisms of sericin proteins, as well as the scavenging and eliminating components remain to be elucidated. Kato *et al* hypothesized that the scavenging function may be provided by the chelating effect of hydroxyl groups of hydroxyamino acids (serine and threonine) that are abundantly contained in sericin (20). Sericin has clearly demonstrated antioxidant capacities against multiple radicals through the measurement of radical scavenging (eliminating) activities by four different methods in this study. Thus, sericin proteins may be efficiently utilized as beneficial for human consumption.

## Figures and Tables

**Table I:** Basic bread formulations.

Ingredient	Amount (g)
Wheat flour	200
Sericin	8
Sugar	13.6
Salt	4
Dry yeast	2.2
Butter	8
Water (tap water)^a^	144

a Water temperature did not exceed 25°C for each sample.

**Table II:** Antioxidant activity of sericin measured by the DPPH-HPLC method.

Sample	Radical-scavenging activity)* (mmol Trolox eq./100 g)
White sericin	18.2±0.7^a^
Yellow-green sericin	23.7±0.4^a^
Bread (no sericin)	3.1±0.5
Bread (added to sericin powder)	3.3±1.4

* Mean ± SD (n=3);

a P<0.001.

**Table III:** Antioxidant activity of sericin measured by the ORAC method.

Sample	ORAC value* (mmol Trolox eq./100 g)
White sericin	10.0±0.4^a^
Yellow-green sericin	29.3±4.0^a^
Bread (no sericin)	0.1±0.0^a^
Bread (added to sericin powder)	0.4±0.0^a^

* Mean ± SD (n=3);

a P<0.05.

ORAC, oxygen radical absorbance capacity.
